# Supplementary figures and images for: Detection and genetic characterization of alphacoronaviruses in co-roosting bat species, southeastern Kenya
Source: PLoS Negl Trop Dis. 2025 Nov 7;19(11):e0012805. doi: 10.1371/journal.pntd.0012805 (PMC12633888; doi:10.1371/journal.pntd.0012805)

## Slide 1
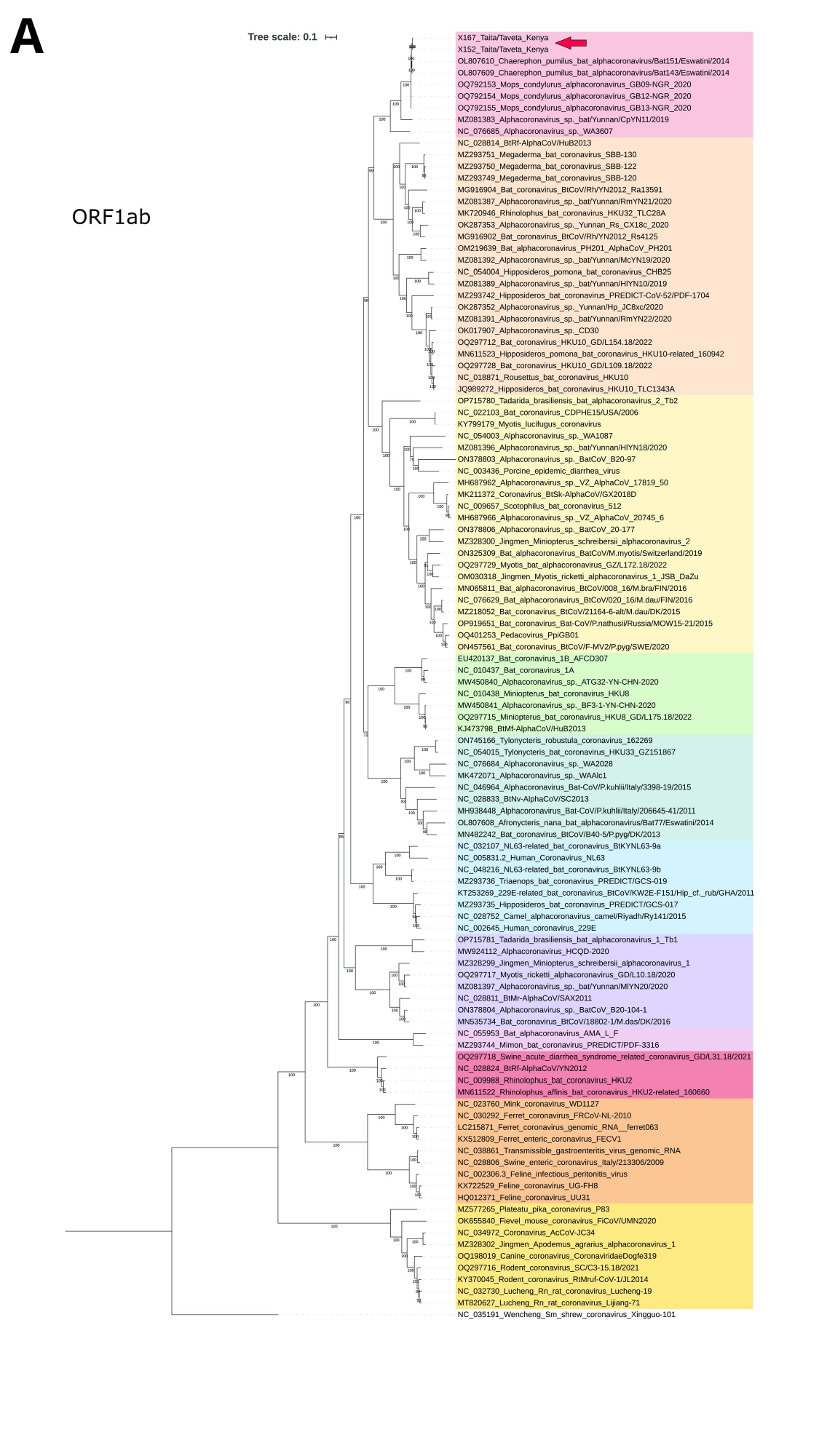

A

## Slide 2
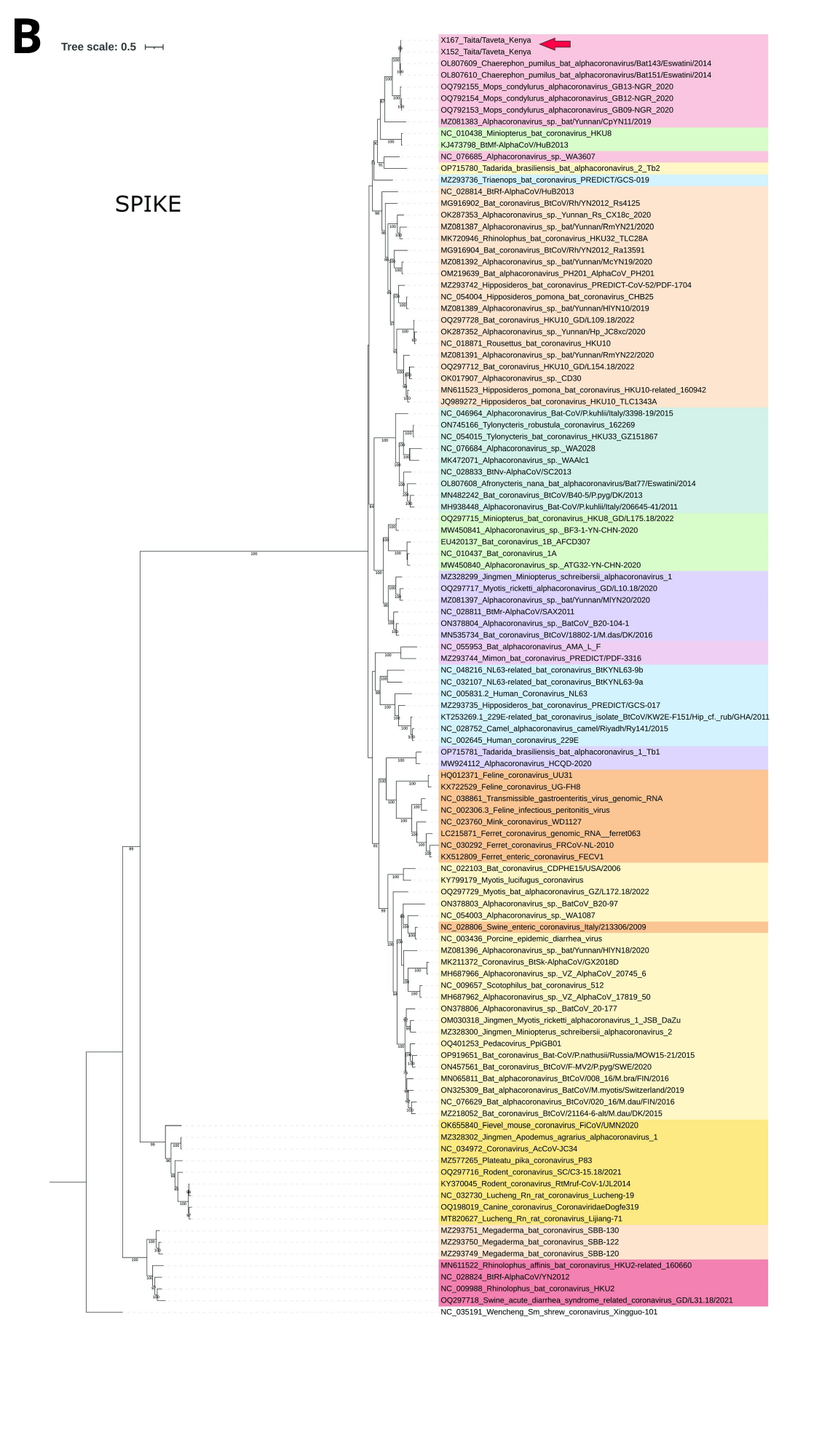

B

## Slide 3
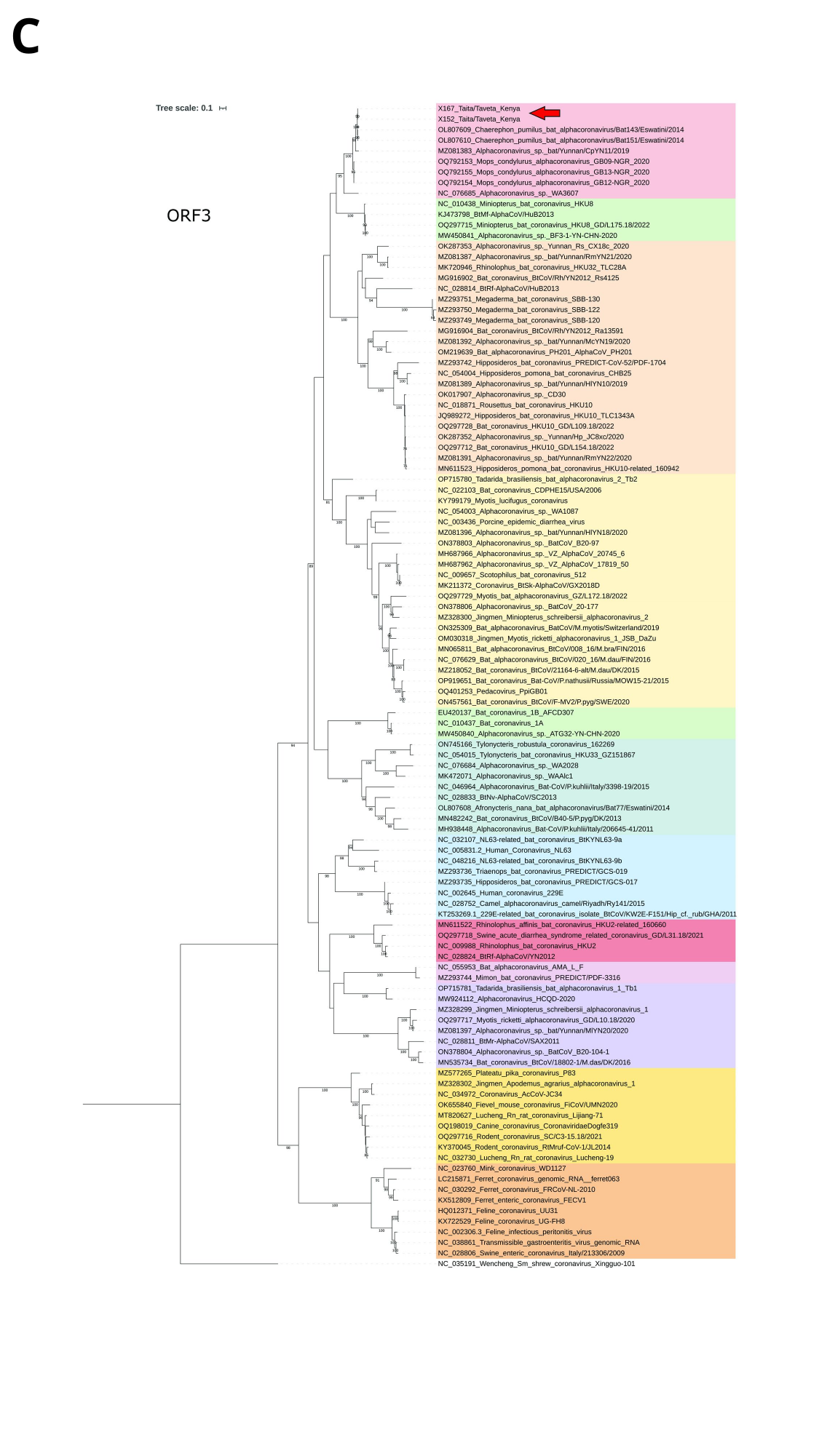

C

## Slide 4
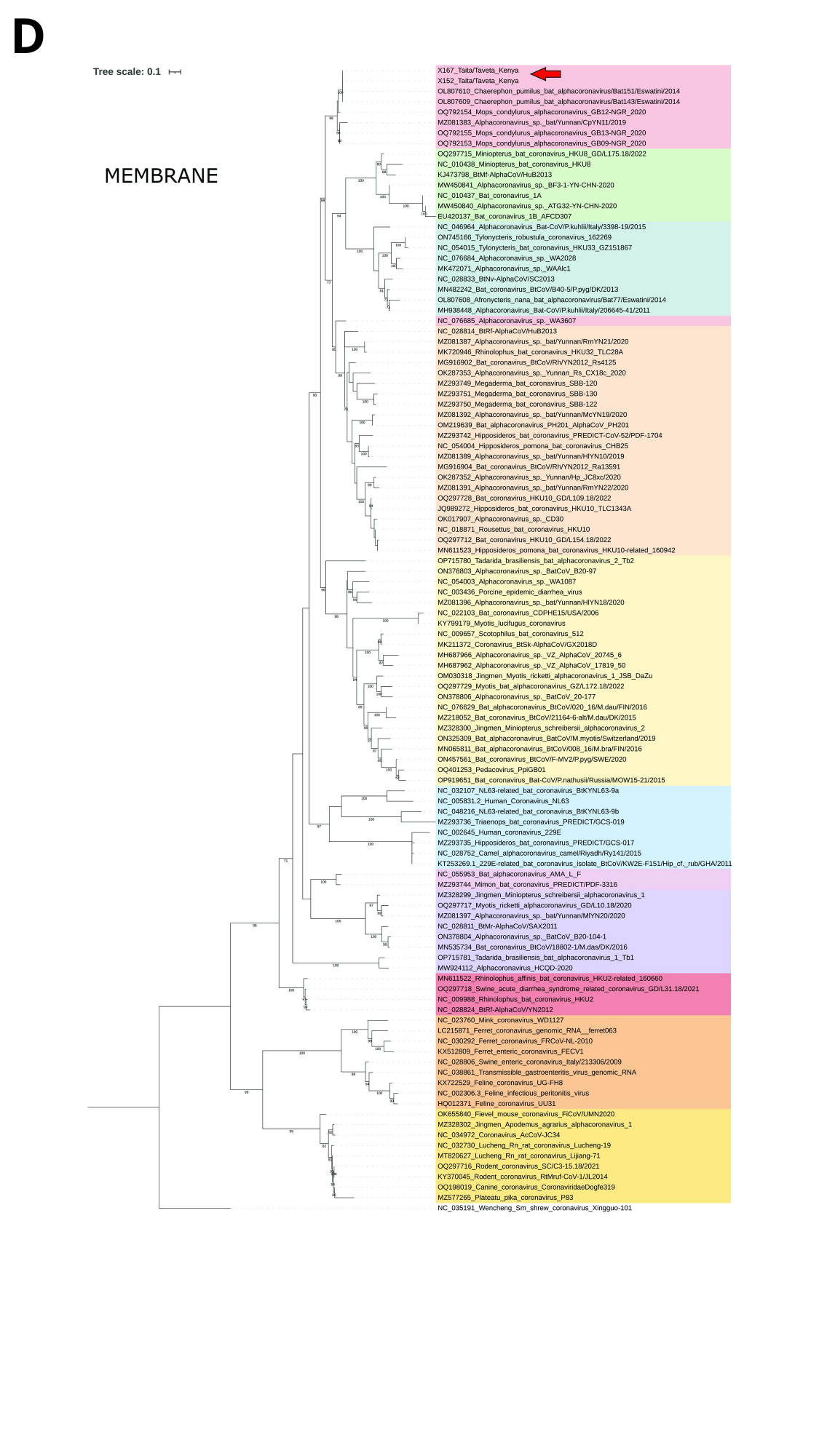

D

## Slide 5
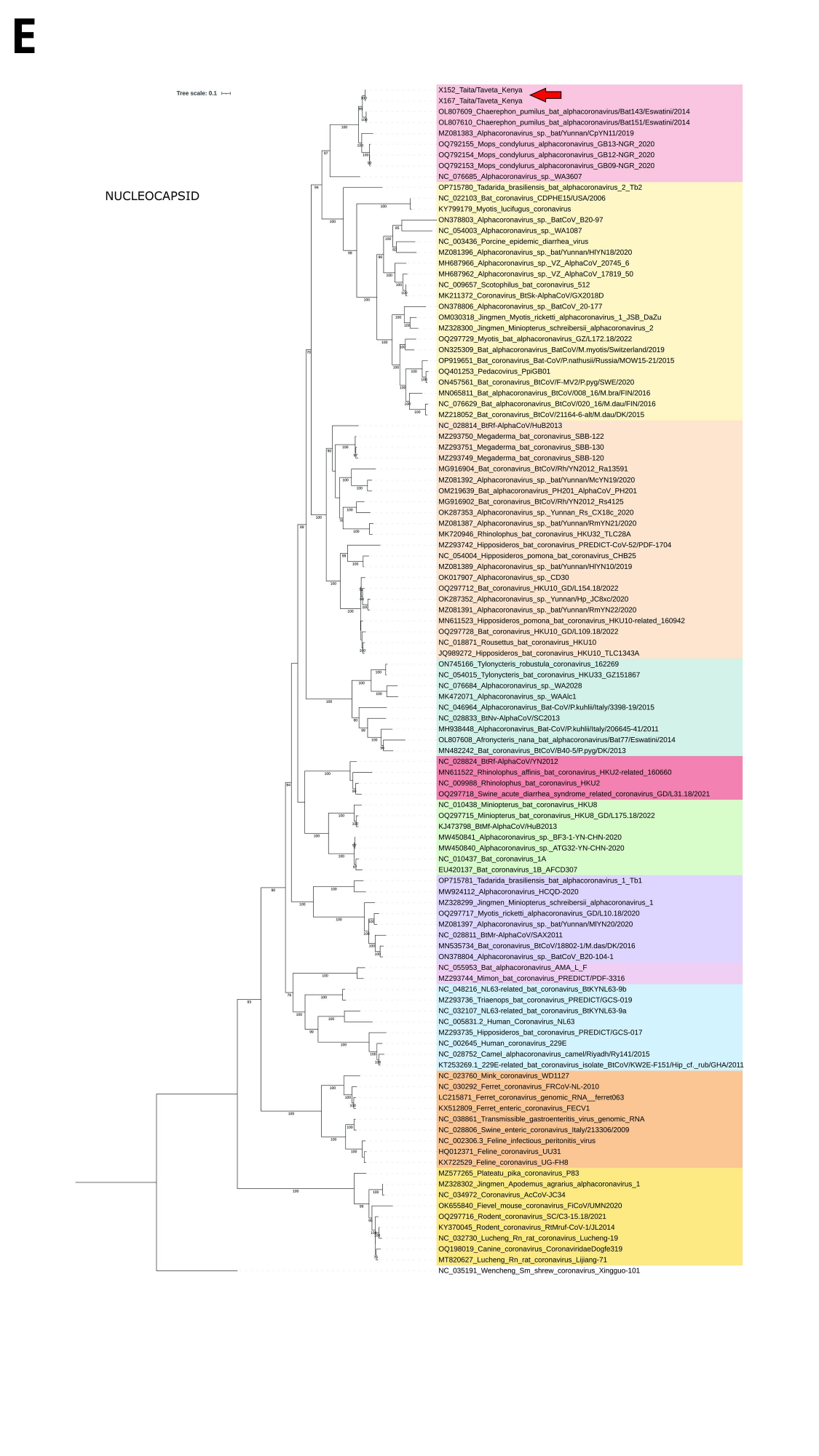

E

Supplement: S1 Fig — A. Phylogenetic trees based on amino acid sequences of the representatives of all available alphacoronavirus species. The trees were constructed with maximum likelihood method implemented in IQTree2 software with best fit substitution models LG + F + R10, WAG + F + R7, LG + F + I + G4, LG + F + I + G4 and LG + F + R5 for ORF1ab (A), spike (B), ORF3 (C), M (D) and N (E) respectively. The clusters are coloured on the basis of ORF1ab clustering pattern. (PPTX) [file pntd.0012805.s001.pptx]
