# Supplementary material for: Detection and genetic characterization of alphacoronaviruses in co-roosting bat species, southeastern Kenya
Source: PLoS Negl Trop Dis. 2025 Nov 7;19(11):e0012805. doi: 10.1371/journal.pntd.0012805 (PMC12633888; doi:10.1371/journal.pntd.0012805)
Supplement: S1 Table — (DOCX) [file pntd.0012805.s002.docx]

**S1 Table.** Summary of sequencing quality and alignment metrics

| **Sample** | **Total Read Count** | **Filtered Read Count** | **Mapped Read Count (MAPQ > 30)** | **Mean Depth** | **Median Depth** |
| --- | --- | --- | --- | --- | --- |
| 152 | 9653282 | 8639236 | 4841583 | 18140.44 | 1184 |
| 167 | 9767360 | 9220686 | 9366590 | 52431.46 | 7271 |
