# Supplementary material for: Detection and genetic characterization of alphacoronaviruses in co-roosting bat species, southeastern Kenya
Source: PLoS Negl Trop Dis. 2025 Nov 7;19(11):e0012805. doi: 10.1371/journal.pntd.0012805 (PMC12633888; doi:10.1371/journal.pntd.0012805)
Supplement: S2 Table — (DOCX) [file pntd.0012805.s003.docx]

**S2 Table.** The results of Hidden Markov Model (HMMER v3.4) search against Pfam database.

|  |  | **full sequence** | | | **best 1 domain** | | |  |  |  |  |
| --- | --- | --- | --- | --- | --- | --- | --- | --- | --- | --- | --- |
| **query name** | **accession** | **E-value** | **score** | **bias** | **E-value** | **score** | **bias** | **start** | **end** |  |  |
| CoV_NSP2_N | PF19211.4 | 3e-84 | 267.8 | 8.9 | 1.1e-83 | 266.0 | 8.9 | 111 | 314 |  |  |
| CoV_NSP2_C | PF19212.4 | 5e-60 | 186.9 | 7.4 | 1.5e-34 | 104.8 | 0.7 | 479 | 591 |  |  |
| CoV_peptidase | PF08715.14 | 1.2e-70 | 224.4 | 8.9 | 4.1e-56 | 176.7 | 2.2 | 1043 | 1229 | 1609 | 1905 |
| CoV_NSP3_C | PF19218.4 | 4.5e-196 | 638.3 | 26.5 | 4.5e-196 | 638.3 | 26.5 | 2024 | 2473 |  |  |
| CoV_NSP4_N | PF19217.4 | 4.5e-132 | 426.5 | 18.7 | 4.5e-132 | 426.5 | 18.7 | 2514 | 2848 |  |  |
| CoV_NSP4_C | PF16348.9 | 8.8e-45 | 137.8 | 2.6 | 4.9e-44 | 135.4 | 2.6 | 2871 | 2966 |  |  |
| Peptidase_C30 | PF05409.17 | 3.9e-128 | 412.8 | 3.7 | 8.4e-128 | 411.7 | 3.7 | 2995 | 3279 |  |  |
| CoV_NSP6 | PF19213.4 | 5.7e-93 | 297.9 | 31.3 | 5.7e-93 | 297.9 | 31.3 | 3293 | 3546 |  |  |
| CoV_NSP7 | PF08716.14 | 1e-39 | 121.4 | 4.9 | 1.3e-39 | 121.0 | 1.7 | 3547 | 3629 |  |  |
| CoV_NSP8 | PF08717.14 | 2.3e-89 | 284.6 | 2.9 | 6e-89 | 283.3 | 2.9 | 3630 | 3821 |  |  |
| CoV_NSP9 | PF08710.14 | 1.7e-45 | 140.3 | 0.0 | 4.4e-45 | 138.9 | 0.0 | 3825 | 3932 |  |  |
| CoV_NSP10 | PF09401.14 | 2.5e-65 | 204.3 | 7.6 | 1e-64 | 202.3 | 7.6 | 3941 | 4062 |  |  |
| CoV_RPol_N | PF06478.17 | 2.6e-183 | 594.8 | 17.1 | 2.6e-183 | 594.8 | 17.1 | 4085 | 4427 |  |  |
| RdRP_1 | PF00680.24 | 1.4e-15 | 42.9 | 0.8 | 2.5e-15 | 42.2 | 0.8 | 4554 | 4845 |  |  |
| CoV_NSP13_ZBD | PF20632.1 | 9.1e-52 | 159.9 | 8.2 | 9.1e-52 | 159.9 | 8.2 | 4995 | 5087 |  |  |
| CoV_NSP13_stalk | PF20633.1 | 9.9e-32 | 95.5 | 1.7 | 4.9e-31 | 93.2 | 1.7 | 5090 | 5137 |  |  |
| CoV_NSP13_1B | PF20631.1 | 1.2e-45 | 140.0 | 0.0 | 4.2e-45 | 138.3 | 0.0 | 5140 | 5222 |  |  |
| Viral_helicase1 | PF01443.22 | 1.9e-11 | 30.3 | 1.5 | 3e-10 | 26.4 | 1.5 | 5273 | 5567 |  |  |
| CoV_ExoN | PF06471.16 | 2e-269 | 880.3 | 16.4 | 3.7e-269 | 879.4 | 16.4 | 5595 | 6104 |  |  |
| CoV_NSP15_N | PF19219.4 | 2.2e-35 | 107.0 | 0.4 | 8.5e-35 | 105.1 | 0.4 | 6110 | 6171 |  |  |
| CoV_NSP15_M | PF19216.4 | 5e-53 | 164.6 | 0.4 | 5e-53 | 164.6 | 0.4 | 6171 | 6293 |  |  |
| CoV_NSP15_C | PF19215.4 | 6.7e-74 | 233.2 | 1.8 | 2.8e-73 | 231.1 | 0.6 | 6293 | 6446 |  |  |
| CoV_Methyltr_2 | PF06460.16 | 6.6e-164 | 530.2 | 5.3 | 1.2e-163 | 529.4 | 5.3 | 6449 | 6747 |  |  |
| CoV_S1 | PF01600.20 | 2.9e-133 | 430.5 | 8.3 | 2.9e-133 | 430.5 | 8.3 | 6962 | 7371 |  |  |
| CoV_S1_C | PF19209.4 | 4.1e-26 | 77.2 | 4.4 | 1.6e-25 | 75.4 | 4.4 | 7386 | 7447 |  |  |
| CoV_S2 | PF01601.20 | 9.3e-233 | 759.5 | 6.3 | 1.8e-232 | 758.6 | 6.3 | 7522 | 8067 |  |  |
| CoV_S2_C | PF19214.4 | 8.9e-13 | 34.7 | 10.9 | 8.9e-13 | 34.7 | 10.9 | 8071 | 8107 |  |  |
| Corona_NS3b | PF03053.18 | 1.2e-61 | 194.4 | 11.4 | 1.2e-61 | 194.4 | 11.4 | 8108 | 8308 |  |  |
| CoV_E | PF02723.18 | 4.2e-27 | 80.5 | 8.3 | 4.2e-27 | 80.5 | 8.3 | 8321 | 8389 |  |  |
| CoV_M | PF01635.22 | 2.1e-88 | 281.2 | 10.6 | 9e-88 | 279.1 | 10.6 | 8405 | 8608 |  |  |
| CoV_nucleocap | PF00937.22 | 9.4e-121 | 389.1 | 32.1 | 2.3e-67 | 213.6 | 17.6 | 8625 | 8991 |  |  |
