# Supplementary material for: Detection and genetic characterization of alphacoronaviruses in co-roosting bat species, southeastern Kenya
Source: PLoS Negl Trop Dis. 2025 Nov 7;19(11):e0012805. doi: 10.1371/journal.pntd.0012805 (PMC12633888; doi:10.1371/journal.pntd.0012805)
Supplement: Attachment — Submitted filename: Ogola Responses to Reviewer 9_6_2025.docx [file pntd.0012805.s005.docx]

**Author Response to Reviewers Comments**

**Dear Michael W. Gaunt (Academic Editor) and reviewers,**

**We are very grateful for the support and thoughtful comments you and the reviewers have provided, which we believe have substantially improved our manuscript. Please find our point-by-point responses below (bolded for clarity).**

**Additional Editor Comments:**
Comments from the AE
Please note that reviewer 2 has questioned the phylogenetics.
The authors originally stated:
"For recombination analysis, a subset of sequences, representing the virus strains that showed the closest homology to those sequenced in this study, were subjected to similarity plot and bootscanning analyses using SimPlot++ software [36] and RDP, GENECONV, BootScan, MaxChi, Chimaera, SiScan, and 3Seq [37–43], as implemented in RDP5 software [44]."

This statement is no longer accurate. It is important to note that phylogenetic incongruence in viruses is not necessarily indicative of recombination. For instance, Gaunt et al. (2022) in Virus Evolution demonstrated that in flaviviruses, strong phylogenetic incongruence can arise not from recombination but from extreme selective pressures. RNA viruses possess exceptional mutational capacities and can undergo reversion under selection pressure, independently generating similar mutations within contiguous loci.

**We have now revised the recombination analysis and stated the limitations of this analysis more explicitly. We agree that any method alone is not sufficient to imply recombination, but by combining both analytical and biological inferences, it is possible to estimate the probability of recombination. While Gaunt et al. (2022) present compelling evidence that recombination does not play a role in (ortho)flavivirus evolution, it is important to note that (ortho)flaviviruses and coronaviruses exhibit markedly different ecological and epidemiological patterns.**

**Specifically, it is likely that coronaviruses show much higher co-infection frequencies than flaviviruses. Coronaviruses have high prevalence among bats, coronavirus transmission occurs directly between mammals via droplets or aerosols, their host species (in this case co-roosting bats) tend to aggregate in large populations and migrate over large distances. Further, bats can shed coronaviruses over relatively long period of time (up to 16 weeks) [Lau et al., 2010] and the infections don’t seem to cause disease in bats, which suggest that infection most likely doesn’t affect the mobility/behavior of infected bats. All these factors favor high frequency of co-infections, which is required for a recombination to occur.**

**Further, here we assessed potential recombination within a single alphacoronavirus species, whereas Gaunt et al., concentrated mainly on *inter-species* recombination. It can be expected that recombination frequency is much higher within a virus species as compared to inter-species recombination frequency, since the recombination within species is less likely to distort the viral protein-protein interactions or cis-acting RNA interactions.**

**Finally, we think that it is unlikely that convergent or parallel evolution would explain the similarities between potentially recombinant loci in our dataset, since these represent nearly identical sequences, including also synonymous mutations.**

**We do agree that the dataset is currently sparse and further studies are required to validate these results. We hope we have adequately discussed this limitation in the revised manuscript (Lines 474-479)**

Additionally, I am not convinced that the manuscript provides evidence of phylogenetic incongruence with the possible small exception of X162. The inference in Figure 2 relies on a bootstrap value of 73 - between Spike and the ORF1/ORF2, which is insufficient for robust conclusions, there would need to be alternative approaches including Bayes and that woudl need to return a robust value for the branch. Figure 3 does indicate some incongruence for X162, but this is not evident in Figure 2. I recommend a thorough reassessment of all phylogenetic incongruence analyses, as the observed signal appears to be a minor feature centered around X162. If the manuscript is resubmitted, I will conduct a full review of all supporting calculations. Also the way the ms is written, it overstating a minor incongruence in a single taxa X162 which only appears present in one analysis.

**We have now reassessed the evidence for recombination, provide a more detailed description of the analysis, discuss the limitations more thoroughly, and added analysis files to increase transparency. The analysis is on Supplementary Tables 1,2,3 for functional domain, recombination analysis for RDP and Robinson-Foulds distance metrics and also Fig 5.**

Moreover, Figure 3 is poorly presented, making it unclear which specific analyses the authors have performed. For instance, bootscanning analysis typically results in a plot, which is absent from the figure. Clarification is needed in this regard. I am not at all clear what the authors have done for Figure 3 and accurate descriptions of all analytics are required to ensure transparency.

**Figure 3 has now been revised and changed to figure 4 with scale**

It is important that the authors resolve the criticisms of their phylogenetic methods and do not overstate their findings because the standards of phylogenetics in PLOS NTD are much higher than the authors have presented.

**We agree that the role of recombination was overstated in the manuscript. We have now revised the description of analyses for clarity and the limitations of the analyses are more clearly stated in the revised version of the manuscript. (Lines 329-378, 474-479)**
The supplementary trees are also missing entirely, which must be rectified. To prevent formatting issues, I strongly recommend submitting these as a PDF.

**This has been appropriately addressed**

Additionally, the table categorizing "Reproductive" and "Non-Reproductive" is confusing for readers unfamiliar with bat biology. Consider rewording or providing additional explanation to ensure clarity.

**The explanation for these categories is provided in the methodology (Lines 111-115), and we have also gone ahead to capture this information on the table heading (Lines 207 -209).**

Finally, I urge the authors to carefully proofread the manuscript for grammatical errors. I can suspect I could produce an extensive list of 50 corrections and if so each would need to be addressed individually.

**The manuscript has been proofread carefully and changes have been made throughout the text.**

**Journal Requirements:**

1) Please ensure that the CRediT author contributions listed for every co-author are completed accurately and in full.
At this stage, the following Authors/Authors require contributions: Joseph Ganda Ogola, Hussein Alburkat, Teemu Smura, Lauri Kareinen, Ravi Kant, Essi M. Korhonen, Tamika J. Lunn, Moses Masika, Paul W. Webala, Philip Nyaga, Omu Anzala, Olli Vapalahti, Kristian M. Forbes, and Tarja A. Sironen. Please ensure that the full contributions of each author are acknowledged in the "Add/Edit/Remove Authors" section of our submission form.

**Appropriately addressed**

The list of CRediT author contributions may be found here: <https://journals.plos.org/plosntds/s/authorship#loc-author-contributions>

2) Please upload all main figures as separate Figure files in .tif or .eps format. For more information about how to convert and format your figure files please see our guidelines:

**Appropriately addressed**
<https://journals.plos.org/plosntds/s/figures>

3) We have noticed that you have uploaded Supporting Information files, but you have not included a list of legends. Please add a full list of legends for your Supporting Information files after the references list.

**Appropriately addressed**

4) Some material included in your submission may be copyrighted. According to PLOSu2019s copyright policy, authors who use figures or other material (e.g., graphics, clipart, maps) from another author or copyright holder must demonstrate or obtain permission to publish this material under the Creative Commons Attribution 4.0 International (CC BY 4.0) License used by PLOS journals. Please closely review the details of PLOSu2019s copyright requirements here: PLOS Licenses and Copyright. If you need to request permissions from a copyright holder, you may use PLOS's Copyright Content Permission form.
Please respond directly to this email and provide any known details concerning your material's license terms and permissions required for reuse, even if you have not yet obtained copyright permissions or are unsure of your material's copyright compatibility. Once you have responded and addressed all other outstanding technical requirements, you may resubmit your manuscript within Editorial Manager. 
Potential Copyright Issues:

- Figure 1. Please provide a direct link to the base layer of the map (i.e., the country or region border shape) and ensure this is also included in the figure legend; and provide a link to the terms of use / license information for the base layer image or shapefile. We cannot publish proprietary or copyrighted maps (e.g. Google Maps, Mapquest) and the terms of use for your map base layer must be compatible with our CC BY 4.0 license.

**Information is added to Figure 1**
Note: if you created the map in a software program like R or ArcGIS, please locate and indicate the source of the basemap shapefile onto which data has been plotted.
If your map was obtained from a copyrighted source please amend the figure so that the base map used is from an openly available source. Alternatively, please provide explicit written permission from the copyright holder granting you the right to publish the material under our CC BY 4.0 license.
If you are unsure whether you can use a map or not, please do reach out and we will be able to help you. The following websites are good examples of where you can source open access or public domain maps:
* U.S. Geological Survey (USGS) - All maps are in the public domain. ([http://www.usgs.gov](http://www.usgs.gov/))
* PlaniGlobe - All maps are published under a Creative Commons license so please cite u201cPlaniGlobe, [http://www.planiglobe.com](http://www.planiglobe.com/), CC BY 2.0u201d in the image credit after the caption. (<http://www.planiglobe.com/?lang=enl>)
* Natural Earth - All maps are public domain. (<http://www.naturalearthdata.com/about/terms-of-use/>).

5) When completing the data availability statement of the submission form, you indicated that you will make your data available on acceptance. We strongly recommend all authors decide on a data sharing plan before acceptance, as the process can be lengthy and hold up publication timelines. Please note that, though access restrictions are acceptable now, your entire data will need to be made freely accessible if your manuscript is accepted for publication. This policy applies to all data except where public deposition would breach compliance with the protocol approved by your research ethics board. If you are unable to adhere to our open data policy, please kindly revise your statement to explain your reasoning and we will seek the editor's input on an exemption. Please be assured that, once you have provided your new statement, the assessment of your exemption will not hold up the peer review process.

**Appropriately addressed**

6) Please ensure that the funders and grant numbers match between the Financial Disclosure field and the Funding Information tab in your submission form. Note that the funders must be provided in the same order in both places as well.
- State the initials, alongside each funding source, of each author to receive each grant. For example: "This work was supported by the National Institutes of Health (####### to AM; ###### to CJ) and the National Science Foundation (###### to AM)."
- State what role the funders took in the study. If the funders had no role in your study, please state: "The funders had no role in study design, data collection and analysis, decision to publish, or preparation of the manuscript.".
If you did not receive any funding for this study, please simply state: u201cThe authors received no specific funding for this work.u201d

**Appropriately addressed**

**Reviewers' Comments:**

Reviewer's Responses to Questions

**Key Review Criteria Required for Acceptance?**

As you describe the new analyses required for acceptance, please consider the following:

**Methods:**

-Are the objectives of the study clearly articulated with a clear testable hypothesis stated?

-Is the study design appropriate to address the stated objectives?

-Is the population clearly described and appropriate for the hypothesis being tested?

-Is the sample size sufficient to ensure adequate power to address the hypothesis being tested?

-Were correct statistical analysis used to support conclusions?

-Are there concerns about ethical or regulatory requirements being met?

Reviewer #1: Methods appear appropriate and well designed.
Some additional details on collection sites would be useful..

**This is added to table 2 (Line 214). The different trapping habitats**

Reviewer #2: I have no ethical concerns related to data collection or sampling. Some statistical problems are addressed in the section about Results below.

Reviewer #3: The study design and methods used are appropriate and well explained.

**Results:**

-Does the analysis presented match the analysis plan?

-Are the results clearly and completely presented?

-Are the figures (Tables, Images) of sufficient quality for clarity?

Reviewer #1: Results are clearly presented and tables are appropriate.

Reviewer #2: In this study, the authors describe sampling of various bat species in southern Kenya, and report on the prevalence of coronavirus RNA sequences among the sampled population. In general, I think there is a great need for work like this, to explore the coronavirus diversity that may one day spill over to other species including humans.

**Thank you for the kind comment**

That said, there are some problems here - I think all of them can be fixed, but it will take some work and will not require additional bat sampling. There is the kernel of a useful paper here, but it could be so much better with additional analysis, and the data need to be much more fully presented.
1. Please add genome maps, showing the parts of each new genome that were recovered. Ideally, this would be done to scale, and at the domain level rather than dividing the genome into overly broad regions like pp1a/pp1ab. New strains often show novelties in some of the accessory genes, and I don't recall a good study on domain-level conservation in the region of pp1a before nsp4 - providing a map of what you found will make this data more useful to the field. For example, I can't tell if you were able to assemble any complete or coding-complete genomes or just partial genomes, and I can't tell how successful your bridging PCR was at closing the gaps. Full or coding-complete genomes can potentially be classified as new species by the ICTV, but from what I understand, much of the material available in the literature is fragmentary to some extent. It would be nice to see your new viruses get the wider attention that I think they deserve.

**We have now included a more detailed description of the genome in the manuscript, including genome maps (Fig 3). Out of the four nearly complete genomes, three (X162, X167 and 193) have complete coding sequences, whereas X152 has two gaps (amino acids 1-42 and 71-114) in ORF1a. We have also included prediction of conserved domains (HMMER v. 3.4 search against Pfam database) (S1 Table). (Line 265, 270)**

**Indeed, we agree that this and other related alphacoronaviruses should be also officially classified and will submit a classification proposal to ICTV in the near future.**
2. Please make the data available and add a table showing the name and accession number for each new genome or fragment. There is potentially some interesting information here about virus transfer within and among species of bats in Kenya. I would very much like to see an attempt at this sort of analysis added to the manuscript.

**We have added a table showing the names and accession numbers for partial sequences and the near complete genomes. Lines 250- 252**

3. Table 1 would be better with male/female stats added. It's fine to keep the reproductive status, but break that down by sex, especially since you appear to be modelling the effect of sex on positivity in table 3. The title seems to suggest sex data was intended to be in this table originally.

**Thank you for this observation, even though we noticed sex was not really a significant predictor for bat coronavirus in this study, we have added sex for the 2-bat species which were of interest. Table 1 (Line 206)**

4. It is not clear what the reference data in Fig. 4 refers to. Data from previous work? Other studies? If there is a source for this data, please cite it in the table, and if this is new data, please describe where/when it was collected.

**Figure 4 (now figure 5) has now been thoroughly revised. (Line 298)**
5. Is the miseq data from these experiments going to be publicly available, and if so, what are the accessions?

**The sequences have already been submitted to genebank with accession numbers available and**

6. In figure 2, what sort of data is being used? Is this amino acid or nucleotide? How long is the aligned contig? What are the boundaries, i.e., does the RdRp here include the NiRAN domain? There is a rather large polytomy in the center, which makes me suspect the data are from a rather narrow region.

**This is 378 nucleotides long alignment that contains** **partial RdRp domain i.e. conserved polymerase motif B in the palm region of RdRp domain (amino acids 614-746 of RdRp (Nsp 12) corresponding to amino acid 4684-4816 of ORF1ab). NiRAN domain is not included. This information has now been added to the revised figure legend. (Line 237-248)**

7. In figure 3, which parts of pp1a are being aligned, and are they homologous? Also please show your new viruses in a different color so they stand out. Same question for pp1b and S - are these parts, entire regions, do they cover important domains? Too many questions unanswered.

**Fig 3 now Fig 4 …..**

**The sequences ON893136 and ON893138 (strains SUOHC3001 and SUOHC1005) are missing 5703 nts from the beginning of the ORF1a and the sequence OM000380 contains only the nucleotide 1539-6893 in the ORF1a. All other strains included in the ORF1a are complete. The sequences included in the phylogenetic trees constructed based on ORF1b and spike are complete in these regions and therefore include all domains. For homology, please see the recombination analysis i.e. one of the sequences does show signs of mosaicism.**

8. In figure 4, are these the only sequenced parts of the genome? The only compared parts? It isn't clear, and two of the parts seem to overlap - does that mean the same data was used twice? The list of confidence values needs a lot more explanation to be useful. Assuming there is a bit of error in the trees due to undersampling and sampling bias, I don't really think any of these trees are different enough in topology to be very conclusive.

**We have now revised the recombination analysis for clarity and also discuss the limitations of the analysis in more detail. Specifically, we first aligned the four complete coding sequences identified in this study (i.e. X152, X162, X167 and X193). Then we identified the potential recombination break points using seven methods implemented in RDP5 software package. The potential recombination events supported by at least five methods (S2 Table) were subjected for further validation. This was done by extracting the breakpoint-free genome regions (named here as BFR-A to BFR-N), and constructing separate phylogenetic trees based on each region followed by assessment of patristic distances between the strains in each tree (Fig 5c) and Robinson-Foulds (RF) distance metrics between the trees (S3 Table)**

**We observed that regions** BFR-B, -D, -F, -H, and -J **had similar tree topologies whereas** BFR-C, -E, and -G **had topologies similar to each other. Only three potential tree topologies were observed (Fig 5c). On the basis of the majority of the ORF1ab -the strain X162 clusters together with X193 whereas X152 clusters together with X167. On some regions, however, X162 diverges from the other sequences, whereas 193 forms a sister taxon for X152 and X167**

**For the two overlapping parts, there indeed was two overlapping recombination signals (i.e. one dataset but overlapping signals). This overlap has now been resolved by re-analyzing the data using only the four strains sequenced in this study, resulting in a clearer signal without overlapping signals for recombination.**

9. There aren't scale bars on any trees - that makes interpretation difficult.

**Scales bars have now been added.**

10. On the supplemental figures, it isn't clear how they relate to Figure 2, or the small trees in Fig. 4 for example. And please color your new viruses or mark them in some way so they can be spotted. Are only these 4 Taita viruses used because they are the most complete? Something like AliStat can be used to give nice statististics relating to the quality of the underlying alignments behind the trees, and there are a number of statistics including TreeDist that would give you a way to compare the similarity or lack thereof between the various trees presented here.

**Alignment stats are now given in Supplementary File**

**Robinson-Foulds distance metrics between consecutive trees are now given in Supplementary File (S3 Table)**

11. Is there evidence, perhaps from other studies, that suggests other types of coronavirus such as Betacoronavirus would have been detected if present? There are some very interesting sarbecoviruses reported from Kenya in other studies, for example.

**Thank you for this question, we have addressed it by including other coronaviruses that has been detected in different bat species in Kenya and indicated that our protocol was broad and would detect any known and novel coronaviruses (Lines 413 - 419)**

**Although we only detected alphacoronaviruses in *M*. *pumilus* and *M*. *condylurus*, alphacoronaviruses and other novel coronaviruses have previously been found in some other bat species in Kenya [49,56]. For example, Tong et al (2006) detected coronaviruses in different species of bats including *Miniopterus inflatus*, *Rousettus aegytiacus* and *Eidolon helvum* and others [63].**

12. A more full discussion of bat sampling in Kenya, Tanzania, Mozambique and throughout eastern Africa would be a welcome addition to the discussion section.

**We have added a discussion on different areas in Kenya, Tanzania, Mozambique and Madagascar where bat coronaviruses have been reported. Line 416 – 418**

Reviewer #3: The results and figures are adequate and clear.

**Conclusions:**

-Are the conclusions supported by the data presented?

-Are the limitations of analysis clearly described?

-Do the authors discuss how these data can be helpful to advance our understanding of the topic under study?

-Is public health relevance addressed?

Reviewer #1: Yes, conclusions appear valid and authors discuss the need for additional surveillance and ultimately gaining an understanding of potential public health relevance.

Reviewer #2: See above - I would need a lot more information to properly judge the conclusions, but on the surface, everything seems reasonable here.

Reviewer #3: The conclusions are supported by the data presented and review of the literature. There could be more information to put these into context, e.g. on the importance of phylogeny of these genomes and on specific gene sequences to their potential to emerge; on the ecology of the bat host species and therefore the likelihood of spillover; and on relationship to alpha-CoVs known to infect livestock.

**Editorial and Data Presentation Modifications?**

Use this section for editorial suggestions as well as relatively minor modifications of existing data that would enhance clarity. If the only modifications needed are minor and/or editorial, you may wish to recommend “Minor Revision” or “Accept”.

Reviewer #1: Minor revision

Reviewer #2: See Results

Reviewer #3: None needed

**Summary and General Comments:**

Use this section to provide overall comments, discuss strengths/weaknesses of the study, novelty, significance, general execution and scholarship. You may also include additional comments for the author, including concerns about dual publication, research ethics, or publication ethics. If requesting major revision, please articulate the new experiments that are needed.

Reviewer #1: The manuscript presented by Ogola and colleagues described molecular surveillance for coronaviruses in bats collected from locations in southeastern Kenya. This is an important problem for study and manuscript is very well written and clearly presented. Similarly, the methods described for detection and analysis of coronavirus sequences appear appropriate and well executed. Overall, I consider this an important contribution to the literature on distribution of bat coronaviruses in Africa. I offer the following small suggestions:

It would be useful to describe the spatial relation between bat capture sites and human habitations. Table 2 lists “buildings”, but were these human dwellings, commercial buildings, hospitals or what? Similarly, please indicate how many geographically distinct sites were subjected to surveillance - a small map of locations in that county would be appropriate but not necessary.

**This is added in the table to indicate which habitats bats were trapped**
Line 23: “Bat species-specific RNA prevalence was ..” – would this be more clear as “The prevalence of detecting coronavirus RNA for specific species of bats was ..”

**Corrected as suggested. Line 24**
Line 42: Suggest changing “Our bat alphacoronaviruses detected..” to “The bat alpha coronaviruses we detected..”

**Corrected as suggested. Line 47-48**
Line 124-6 describes the PCR primers used to detect CoV. The reference is almost 20 years old and, if appropriate, it would be good to add a comment that these primers are known to detect all 4 genera of coronaviruses currently known (that is, we’ve learned a lot about new coronaviruses over since 2007 and it would be confirm those primers will not miss detection of recently discovered coronaviruses)

**We used a pan-coronavirus assay that can detect both known and novel coronaviruses, and we have added this statement (Lines 150 - 151, 413-416). Despite all the development since its publication, this method is still excellent when searching widely for novel coronaviruses. We did specify in the text that this method is particularly good for bats, as we have seen in our yet unpublished research that this method is not equally good for all mammal species.**

Reviewer #2: See Results. There is the kernel of a very nice study here, but the presentation needs lots of work before it can be completely reviewed.

Reviewer #3: Major comments
Overall this is a solid paper reporting whole genomes from some interesting alpha-CoVs in Kenya. The discussion could be expanded and made more informative by reference to the following:
• Some information on the likely ability of these new CoVs to infect people (from genome data/phylogeny)

**The viruses described in this manuscript are members of decacovirus subgenus and to our knowledge, none of the members of this subgenus have ever been found in humans. However, since both the virus and the bat host are abundant, such a host-jump is a concern and therefore we think that these viruses should be monitored closely.**

• Information on ecology of bat host species that might have relevance for spillover

**We have added a paragraph on the ecology of bats that increases the likelihood of spillover of the CoV they host. Lines 420 – 430**

**We detected coronaviruses in bats trapped from houses occupied by humans and also water points which are used by both humans and livestock for drinking. This poses a significant zoonotic risk for the spillover of bat borne zoonotic viruses, particularly coronaviruses which have been documented by studies across Africa (Kumakamba et al., 2021; Gibb et al., 2022). In most rural communities in Africa, molossid bats, in particular *M*. *pumilus* and *M*. *condylurus* often roost in human occupied buildings, including roofs, ceilings and wall cavities, increasing contact with humans and livestock. Livestock in particular may act as an intermediate or amplifying host. Such close interaction increases the likelihood of viral exposure through contamination with bat excreta, saliva, or urine. In addition to the low public awareness of zoonotic risks, the risk of exposure is amplified in rural communities due to limited resources and housing conditions that may be inadequate to prevent wildlife invasion.**

• Reference to the ability of other alpha CoVs to infect livestock, recently (e.g. SADS-CoV) or historically (reviewed in various papers by Saif & most recently in PNAS Keusch et al.)

**In the conclusion statement, we have highlighted the recent example of livestock associated spillover of bat associated alphacoronavirus to livestock. Line 483 – 485**

**Severe acute diarrhea syndrome coronavirus (SADS-CoV), an emerging virus responsible for severe, acute diarrhea in piglets in China is one of the most recent bat-derived alphacoronavirus.**

Minor comments
L 64 – should be ‘likely zoonotic’ – I think they probably are but not yet definitively proven

**Corrected as suggested. Line 73**

L69 – these are just some of the reasons for zoonotic spillover of CoVs – others are ecological/demographic – human interactions with bats, environmental changes like deforestation and the unsustainable wildlife trade.

**Corrected as suggested. Line 78**

PLOS authors have the option to publish the peer review history of their article ([what does this mean?](https://track.editorialmanager.com/CL0/https:%2F%2Fjournals.plos.org%2Fplosntds%2Fs%2Feditorial-and-peer-review-process%23loc-peer-review-history/1/010f01957f69ddfc-7ff2aa96-fb6d-4a04-93da-794863ceb769-000000/kTpRSLB8b60NvuNNVhsYx_4AOWi83r5yDgYDlAmyYmw=201)). If published, this will include your full peer review and any attached files.

Thank you

Sincerely,

Joseph Ogola

On behalf of other co-authors
