# Supplementary material for: Detection and genetic characterization of alphacoronaviruses in co-roosting bat species, southeastern Kenya
Source: PLoS Negl Trop Dis. 2025 Nov 7;19(11):e0012805. doi: 10.1371/journal.pntd.0012805 (PMC12633888; doi:10.1371/journal.pntd.0012805)
Supplement: Attachment — Submitted filename: Ogola Response to Reviewers .docx [file pntd.0012805.s006.docx]

**Author Response to Reviewers Comments**

**We are very grateful for the support and thoughtful comments you and the reviewers have provided, which we believe have substantially improved our manuscript. We have also excluded recombination analysis from the manuscript. Please find our point-by-point responses below (bolded for clarity).**

**Additional Editor Comments:**

**# AE Editorial Note on Recombination Claims and Phylogenetic Analysis**

Both Reviewer 2 and I continue to have serious concerns regarding the authors’ phylogenetic analyses, which represent a demonstrable weakness in the manuscript. This directly affects the validity of the authors' claims, especially the assertion in the Abstract:

> "Incongruent clustering patterns based on distinct genomic regions suggest that this virus may have undergone recombination events during its evolution."

Despite previous feedback, the authors continue to assert recombination without sufficient supporting evidence. This claim must be removed unless a substantially stronger analytical foundation is provided.

1. Lack of Evidence for Recombination

The authors have relied on RDP software but appear to have used only a subset of available methods. They have excluded key approaches within RDP that account for stochastic noise, such as those that handle variation at the third codon position. The analysis is further weakened by reliance on incongruent trees over small genetic distances (few mutations) that are not convincing and a sliding window similarity analysis (Fig. 5b) without appropriate correction.

Specifically, the use of raw nucleotide "similarity" as a metric (described on the y-axis in Fig. 5b) is outdated and introduces known artefacts. This issue was addressed over 50 years ago by Jukes and Cantor (1969), whose correction is foundational in modern molecular phylogenetics. The authors’ approach in Fig. 5b disregards this standard and constitutes a major methodological flaw.

2. Authors’ Justifications – Inadequate and Misapplied

The authors’ defence of their recombination claim is based on three points:

a. Multi-clonality
The sequencing and assembly approach used assumes clonality, yet the authors now claim evidence of multi-clonal viral populations. However, no bioinformatic or biological methods were employed to remove/separate mixed infections or identify haplotypes. It is far more likely that the apparent signal of recombination stems from assembly artefacts caused by mixed reads than from biological recombination. Quality control metrics for the NGS data are also lacking.

b. Intra-species Sampling; see point 3

c. Coronaviruses are “known” to recombine
Line 291 of the manuscript states:

> “Since coronaviruses are known for their capacity for homologous recombination \[62]...”

While recombination has been documented in coronaviruses, the frequency of such events in natural populations remains low. For example, despite billions of SARS-CoV-2 infections, confirmed recombinants are limited. The biological capacity for recombination does not justify claiming its presence in the absence of compelling evidence in the specific dataset.

3. Intra-species Sampling
The authors argue that recombination is far more likely within a single species. However, this claim is both historically and methodologically weak. A relevant example is dengue virus: serotypes of dengue (now considered distinct species) produced strong signals of phylogenetic incongruence in early analyses, and these results were published in high-impact journals over two decades ago. These early findings even contributed to the development of recombination detection tools that rely on incongruent tree topologies.

Yet despite this historical precedent, the field has since shifted. Notable leading researchers — including Vasilakis and Weaver — now argue that dengue virus does not, in fact, recombine, or does so only rarely. This illustrates the danger of prematurely labeling incongruent signal as recombination: once such claims are in the literature, they are difficult to retract or revise, even as analytical tools and interpretations evolve. The authors' current data and methods fall below those early dengue virus studies, making their recombination claims concerning.

4. The Broader Implication

The current manuscript presents weak and methodologically outdated evidence to support a claim of recombination. If published, this would lower the analytical standards of the field and risk reinforcing inaccurate evolutionary narratives. Indeed, the star-like phylogeny in Fig. 2 might itself result from unresolved multi-clonal signals, further complicating interpretation.

5. Required Action

The authors must:

Remove all claims of recombination unless they are supported by robust phylogenetic and statistical evidence.

**We re-assessed the clonality of the consensus sequences by calling the minority variants using LoFreq (Wilm et al., 2012); details of sequence quality control are provided below. Two samples (X152 and X167) exhibited only minor intra-sample variation, with all minority variants below 10% frequency and most under 1% frequency, consistent with transient or stochastic intra-host variation observed in coronaviruses. In contrast, samples X162 and X193 showed substantially higher intra-sample variation with many variants in the 20-80% frequency range. Given that these data were generated using short-read sequencing, consensus calling from such assemblies is not sufficiently reliable to support conclusions about recombinant origin. Accordingly, we have removed samples X162 and X193 from the analysis, revised the manuscript and removed the recombination analysis.**

Deposit raw NGS reads and associated metadata in a public repository (e.g., NCBI SRA).

**The raw NGS reads for this study have been deposited in the European Nucleotide Archive (ENA) at EMBL-EBI under accession number PRJEB96286 (Lines 242 – 243)**

Provide appropriate quality control metrics and explain assembly parameters, particularly with respect to potential mixed infections.

**We have now added a more detailed description of the quality control procedures for read filtering and assembly in the Methods section. Specifically, raw reads were quality-filtered using the following parameters: a base quality score threshold of 30, a maximum of 40% low-quality bases per read, a minimum read length of 25 bp, and a sliding window of 20 bp with a mean quality threshold of 30. Base correction for paired-end reads was also enabled. To ensure high-confidence mapping, reads were further filtered by mapping quality (MAPQ ≥ 30) prior to consensus calling. (Lines 173 – 182)**

**A summary of sequencing quality and alignment metrics is now provided in S1Table (Line 184)**

**These parameters were selected to minimize sequencing noise and reduce the likelihood of false-positive variant calls, particularly in the context of potential mixed infections. Minority variant analysis was performed separately to assess intra-sample diversity and clonality (see above).**

This is the final opportunity to address these issues. If the current language and analyses remain unchanged, the manuscript risks rejection on grounds of methodological inadequacy and potential to mislead.

**Journal Requirements:**

1) Please upload all main figures as separate Figure files in .tif or .eps format. For more information about how to convert and format your figure files please see our guidelines: 
<https://journals.plos.org/plosntds/s/figures>

2) Please amend your detailed Financial Disclosure statement. This is published with the article. It must therefore be completed in full sentences and contain the exact wording you wish to be published.
1) State what role the funders took in the study. If the funders had no role in your study, please state: "The funders had no role in study design, data collection and analysis, decision to publish, or preparation of the manuscript."
2) If any authors received a salary from any of your funders, please state which authors and which funders..
If you did not receive any funding for this study, please simply state: u201cThe authors received no specific funding for this work.u201d

3)  Please ensure that the funders and grant numbers match between the Financial Disclosure field and the Funding Information tab in your submission form. Note that the funders must be provided in the same order in both places as well.

**Reviewers' Comments:**

Reviewer's Responses to Questions

**Key Review Criteria Required for Acceptance?**

As you describe the new analyses required for acceptance, please consider the following:

**Methods**

-Are the objectives of the study clearly articulated with a clear testable hypothesis stated?

-Is the study design appropriate to address the stated objectives?

-Is the population clearly described and appropriate for the hypothesis being tested?

-Is the sample size sufficient to ensure adequate power to address the hypothesis being tested?

-Were correct statistical analysis used to support conclusions?

-Are there concerns about ethical or regulatory requirements being met?

Reviewer #1: The authors have done a good and appropriate job of responding to reviewer comments, adding clarity to several issues on phylogenetic and ecological analysis.

**Thank you for the comment**

Reviewer #2: Open questions – how are the trees rooted? It seems like there may be different answers for Figs. 2, 4 and 5.

**The trees in Fig 2 and Fig 4 are rooted to midpoint. Figure 5 has been removed from the revised manuscript. This information has now been added to the manuscript (Lines 260 and 315).**

What were the criteria for showing polytomies as opposed to distinct branches – it seems like a rule of some sort was being applied, maybe by default, and possibly sensibly, but it would help me to see it articulated.

**The nodes with bootstrap support less than 70 were polytomized, since the bootstrap support values below 70% are commonly considered unreliable (Hillis, D.M. and Bull, J.J. (1993) An Empirical Test of Bootstrapping as a Method for Assessing Confidence in Phylogenetic Analysis. Systematic Biology, 42, 182-192). This information has now been added to the manuscript (Lines 261)**

**Results**

-Does the analysis presented match the analysis plan?

-Are the results clearly and completely presented?

-Are the figures (Tables, Images) of sufficient quality for clarity?

Reviewer #1: Yes, analysis is well described.

Reviewer #2: Presentation is not particularly slick, but does what the authors and reader need, I think.

**Conclusions**

-Are the conclusions supported by the data presented?

-Are the limitations of analysis clearly described?

-Do the authors discuss how these data can be helpful to advance our understanding of the topic under study?

-Is public health relevance addressed?

Reviewer #1: Conclusions appear valid

Reviewer #2: The conclusions seem reasonable to me, with the possible exception of the recombination analysis.

**Editorial and Data Presentation Modifications?**

Use this section for editorial suggestions as well as relatively minor modifications of existing data that would enhance clarity. If the only modifications needed are minor and/or editorial, you may wish to recommend “Minor Revision” or “Accept”.

Reviewer #1: No issues noted

Reviewer #2: I’m not sure how robust or useful the recombination analysis is. I defer to the other reviewer’s expertise on that. If it was me, I’d probably delete panels C and D and the corresponding supplementary information. It’s just a matter of having very thin data for the whole-genome recombination analysis – these trees have 8 or fewer nodes, 5 or fewer if you factor in polytomies and near-identical sequences – and I don’t think there is enough signal there for a meaningful RF analysis of the resulting trees.

**We have completely excluded recombination analysis**

I also suspect BFR-C and BFR-F are picking up known hypervariable regions of nsp2 and nsp3, respectively, rather than genuine recombinant breaks.

**Figure 5 has been completely removed in the manuscript**

**Summary and General Comments**

Use this section to provide overall comments, discuss strengths/weaknesses of the study, novelty, significance, general execution and scholarship. You may also include additional comments for the author, including concerns about dual publication, research ethics, or publication ethics. If requesting major revision, please articulate the new experiments that are needed.

Reviewer #1: This manuscript has been significantly improved and provides a valuable contribution to the study of bat coronaviruses.

**Thank you for the comment**

Reviewer #2: This is better than the last time I saw it. A quick blast against named coronavirus species shows that four most complete sequences (X193, X152, X162 and X167) all likely belong to the same species, which is distinct from previously described species, which adds to the value of the study. They do fit into a known subgenus, by the look of it, as the authors say, but it has a surprisingly broad global distribution, which I think is interesting.

The addition of what will likely be a new species is still the major selling point for me, as is the co-roosting species sampling design. The grouping of sequences by host species in Fig. 2 would seem to show more evidence that cross-species transmission is rare, with longer periods of host-specific adaptation between rare species jumps, despite some degree of co-roosting. Mostly, I like that you looked at this question.

**Thank you for this observation, we plan to address and answer this interesting question in our ongoing bat surveillance in Kenya**.

Sincerely,

Joseph Ogola

On behalf of other co-authors
